# A Physics-Aware Diffusion Framework for Robust ECG Synthesis Using Mesoscopic Lattice Boltzmann Constraints

**DOI:** 10.3390/biology15050431

**Published:** 2026-03-05

**Authors:** Xi Qiu, Hailin Cao, Li Yang, Hui Wang

**Affiliations:** 1 College of Mechanical and Vehicle Engineering, Chongqing University, Chongqing 400044, China; 20210962@cqu.edu.cn; 2 School of Microelectronics and Communication Engineering, Chongqing University, Chongqing 400044, China; 202412021102t@stu.cqu.edu.cn (L.Y.);

**Keywords:** hemodynamics, photoplethysmography, electrocardiography, physics-informed deep learning, cardiovascular monitoring

## Abstract

Cardiovascular disease has become the leading cause of death worldwide, underscoring the urgent need for widespread cardiac monitoring, while the Electrocardiogram (ECG) remains the diagnostic gold standard, the complexity of its acquisition limits its long-term feasibility. In contrast, Photoplethysmography (PPG), ubiquitous in wearable devices, is increasingly adopted due to its accessibility. However, synthesizing ECG from PPG poses an intrinsically ill-posed inverse problem. Existing purely data-driven paradigms often neglect underlying biophysical mechanisms, resulting in a lack of physical constraints and interpretability, which renders them prone to generating non-physiological hallucinations. To address this, we propose PhysDiff-LBM, a novel physics-aware framework that incorporates Lattice Boltzmann hemodynamic constraints into a conditional diffusion model. Employing a dual-stream architecture, our framework captures high-frequency morphological details via a cross-attention-guided diffusion model with region-wise adaptability. Synergistically, we physically regularize the ECG synthesis by leveraging the mesoscopic streaming and collision operators of LBM. By forcing the synthesized waveform gradients to evolve consistently with hemodynamic momentum, this mechanism constrains the model to strictly adhere to the fluid dynamic conservation laws governing pulse wave propagation. Experimental results demonstrate that our method achieves superior signal fidelity and exhibits significant advantages in downstream clinical applications.

## Simple Summary

Smartwatches and fitness bands track our pulse using simple optical sensors, but diagnosing true heart disease requires an electrical electrocardiogram (ECG) typically recorded in hospitals with sticky patches and wires, while artificial intelligence (AI) has been used to translate simple wrist pulse data into clinical ECGs, standard AI often “guesses” the waveforms, creating medically incorrect or physically impossible results. To overcome this, we developed a new AI system that directly embeds the natural physical rules of blood circulation into its learning process. Instead of just learning from data patterns, we taught the AI the actual physical laws of how blood pumps from the heart and flows through blood vessels. Constrained by these natural laws of fluid dynamics, our AI is prevented from making impossible physiological guesses. Our tests show this physics-guided approach successfully turns ordinary pulse data into highly accurate, doctor-ready ECGs, bringing us one step closer to hospital-level heart monitoring directly from everyday wearables.

## 1. Introduction

Cardiovascular diseases (CVDs) remain the leading cause of mortality globally, necessitating continuous and ubiquitous cardiac monitoring [1], while the Electrocardiogram (ECG) stands as the clinical gold standard for diagnosing cardiac arrhythmias and myocardial anomalies [2,3], its acquisition typically requires adhesive electrodes and professional operation, limiting its utility for long-term home monitoring. In contrast, Photoplethysmography (PPG) has become ubiquitous in wearable devices due to its non-invasive and low-cost nature [4,5]. Consequently, the cross-modal synthesis of ECG signals from PPG—effectively translating peripheral blood volume changes back to cardiac electrical activity—has emerged as a pivotal research frontier in AI-driven healthcare [6,7,8]. Fundamentally, the feasibility of such estimation is rooted in the physiological principle of cardiac electromechanical coupling [9,10,11]. The electrical excitation of the heart (captured by ECG) triggers the mechanical contraction of the myocardium, which subsequently generates a pulsatile pressure wave propagating through the vascular tree (captured by PPG). Consequently, the PPG signal inherently embeds latent information regarding the cardiac electrical cycle. However, retrieving the ECG from PPG is non-trivial; the vascular system acts as a biological low-pass filter, dampening the high-frequency components associated with sharp electrical transitions [12,13,14]. Despite this, the intrinsic correlation between these modalities suggests that with advanced modeling, wearable PPG can serve as a viable surrogate for reconstructing high-fidelity ECGs, thereby significantly expanding the clinical utility of consumer-grade devices for ambulatory cardiac monitoring [15].

Technically, reconstructing ECG from PPG is an ill-posed inverse problem, as the mapping from peripheral hemodynamic signals to cardiac electrical signals is complex, non-linear, and subject to individual variability. Early approaches primarily relied on signal processing techniques or shallow machine learning models, which struggled to capture the intricate temporal dependencies of physiological signals [16,17]. With the advent of deep learning, generative models such as Generative Adversarial Networks and standard Variational Autoencoders have demonstrated improved performance [18,19]. More recently, Denoising Diffusion Probabilistic Models have achieved remarkable success in various generative tasks due to their stable training dynamics and high sample quality. These attributes position them as a promising candidate to overcome the limitations of GANs and VAEs, offering immense potential for capturing the complex, non-linear mappings essential for high-fidelity physiological signal reconstruction [20,21].

**Figure 1:**
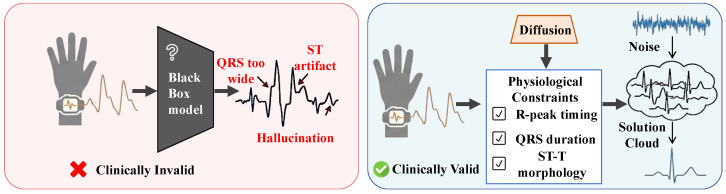
Comparison of black-box models versus physics-constrained diffusion for PPG-to-ECG translation.

However, as illustrated in Figure 1, existing data-driven approaches, including standard diffusion models, face significant limitations in biomedical domains. They tend to prioritize statistical correlation over physiological plausibility [22,23]. Without explicit physical constraints, these models often suffer from “hallucinations”—generating waveforms that may appear visually realistic but violate fundamental hemodynamic principles or lack clinical consistency in critical regions, such as distorted QRS durations or physically impossible repolarization patterns. Furthermore, pure data-driven models often struggle to balance the reconstruction of low-frequency trends governed by hemodynamics and high-frequency details reflecting electrophysiological nuances [24,25].

To mitigate these issues, we argue that the generation process should be guided by the underlying physics of blood flow. In this paper, we propose PhysDiff-LBM, a novel physics-aware framework that rigidly incorporates hemodynamic constraints into a conditional diffusion model. Our approach augments the time-series prediction of pulse wave propagation by integrating a mesoscopic particle streaming and collision process governed by the Lattice Boltzmann Method (LBM).

Specifically, we design a unique dual-stream architecture. The first stream employs a cross-attention-guided diffusion model equipped with region-wise adaptability. This mechanism allows the model to adaptively focus on and refine high-frequency details in critical cardiac phases while maintaining global coherence. The second stream integrates a differentiable LBM solver that simulates the fluid dynamics of pulse propagation, ensuring the generated signals adhere to conservation laws. These two components are synergistically coupled to enforce physical consistency between the electrical and hemodynamic domains, effectively suppressing non-physiological artifacts. The main contributions of this work are summarized as follows:

(1) We propose PhysDiff-LBM, the first framework to our knowledge that integrates Lattice Boltzmann hemodynamic constraints with diffusion models for ECG synthesis, effectively bridging the gap between data-driven generation and physical modeling.

(2) We introduce a region-aware cross-attention mechanism within the diffusion backbone, enabling the model to capture high-frequency morphological details with local adaptability, significantly enhancing the fidelity of critical waveform segments.

(3) We develop a differentiable LBM solver to impose physics-informed constraints, thereby enforcing hemodynamic plausibility and reducing the occurrence of hallucinations common in pure deep learning approaches.

(4) Extensive experiments, comprising quantitative benchmarks against leading competitive baselines, qualitative visual analysis, and downstream clinical evaluations, demonstrate the superiority of our framework. Our method not only achieves significantly improved accuracy in reconstruction metrics but also preserves high-fidelity morphological details and exhibits robust performance in practical cardiac diagnostic tasks.

## 2. Method

### 2.1. Problem Formulation

**Figure 2:**
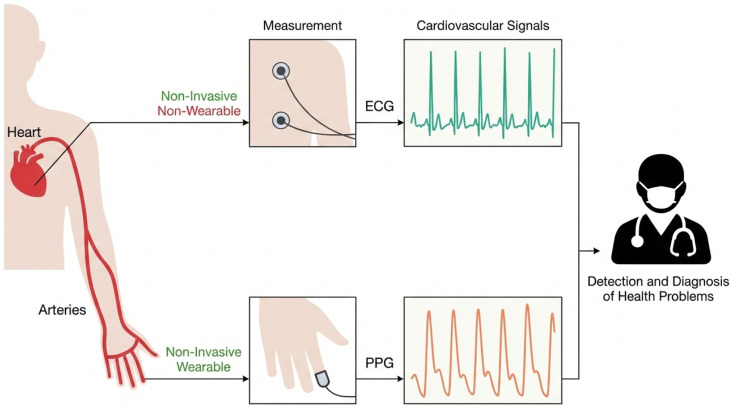
Illustration of cardiovascular signal measurement paradigms. ECG captures cardiac electrical activity directly from the chest, whereas PPG measures peripheral arterial blood volume changes. This study aims to bridge the domain gap to reconstruct ECG from PPG.

The primary objective of this study is to reconstruct high-fidelity Electrocardiogram (ECG) signals from single-lead Photoplethysmography (PPG) recordings (see Figure 2). Let $c\in R^{L}$ denote the observed input PPG sequence of length *L*, and $x_{0}\in R^{L}$ denote the corresponding ground-truth ECG signal. The relationship between PPG and ECG is governed by complex physiological coupling—specifically the interaction between cardiac electrical excitation and peripheral hemodynamic propagation—rendering the inverse mapping $F:c\to x_{0}$ a highly ill-posed problem with non-unique solutions.

We formulate this reconstruction task as a conditional generative modeling problem. Our goal is to learn the conditional distribution $p_{\theta }\left(x_{0}|c\right)$, enabling the synthesis of ECG waveforms that are not only morphologically accurate but also physically consistent with the underlying hemodynamic principles extracted from the PPG modality.

**Figure 3:**
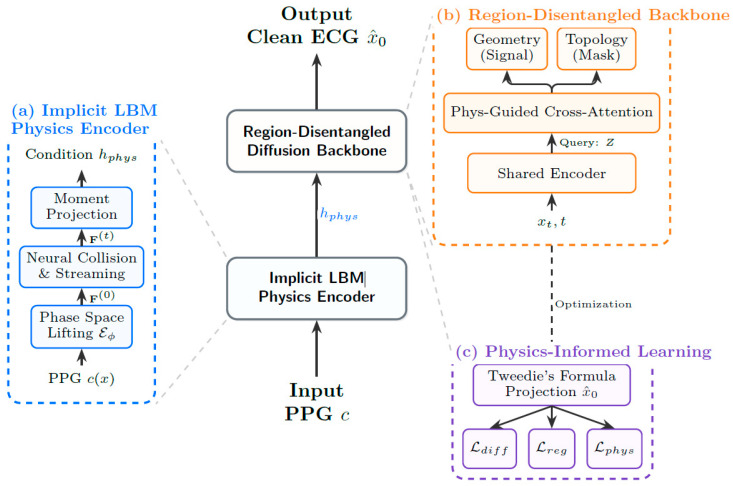
The Overall Architecture of Phydiff-LBM.

To address the ill-posed nature of this cross-domain inverse problem, we propose **PhysDiff-LBM**, a physics-informed generative framework illustrated in Figure 3. The system pipelines three synergistic components to enforce physiological fidelity across domains. First, the **Implicit LBM Physics Encoder** (Section 2.2) lifts macroscopic PPG signals into a mesoscopic phase space, simulating blood flow dynamics to extract robust hemodynamic invariants. This physical context then conditions the **Region-Disentangled Diffusion Backbone** (Section 2.3), a dual-stream architecture that synthesizes the ECG signal by jointly optimizing for fine-grained morphological geometry and global topological rhythm. Finally, to rigorously bind the generation to physical laws, our **Physics-Informed Generative Learning** strategy (Section 2.4) employs Tweedie’s formula to integrate fluid dynamic constraints directly into the diffusion training objective, ensuring the synthesized waveforms maintain strict hemodynamic consistency.

### 2.2. Implicit Lattice Boltzmann Physics Encoding

#### 2.2.1. Bridging Hemodynamics and Kinetic Theory

The physiological link between PPG and ECG is fundamentally mediated by the cardiovascular system’s hemodynamic response. The heart’s electrical activation triggers a mechanical pressure wave that propagates through the vascular tree. Classically, this blood flow dynamics is governed by the conservation laws of mass and momentum, formalized as the Navier–Stokes equations for incompressible fluid [26]:

(1) $$\begin{array}{cc} \nabla \cdot u & =0,\\ \rho \left(\frac{\partial u}{\partial t}+\left(u\cdot \nabla \right)u\right) & =-\nabla p+\mu \nabla^{2}u+f, \end{array}$$

where ρ denotes blood density, u is the flow velocity vector, *p* is the pressure, μ represents dynamic viscosity, and f denotes external body forces. Additionally, ∇ and $\nabla^{2}$ denote the gradient and Laplace operators, respectively.

While Equation (1) provides a rigorous macroscopic description, directly embedding it as a prior in generative modeling imposes significant limitations. The N-S formulation models fluid motion through *averaged* macroscopic variables, effectively assuming local thermodynamic equilibrium [27]. In the context of deep generative learning, enforcing these macroscopic constraints acts as an aggressive low-pass filter. The neural network is forced to prioritize the smoothness of the pressure field to satisfy the partial differential operators, often at the expense of high-frequency signal components. Consequently, N-S based approaches tend to generate overly smoothed waveforms, failing to capture the stochastic, sharp morphological details that are critical for clinical diagnosis.

To overcome this “spectral bias,” we resort to Kinetic Theory. Specifically, we adopt the *Lattice Boltzmann Method (LBM)*, which describes the fluid not by macroscopic variables, but by the particle distribution function f(x,ξ,t), representing the probability of finding a particle with continuous microscopic velocity ξ at position x and time *t*. In the discrete LBM formulation, the continuous velocity ξ is reduced to a finite set of *Q* lattice vectors $e_{i}$, effectively absorbing the velocity parameter into the subscript to form the discrete distribution $f_{i}\left(x,t\right)$. The evolution of $f_{i}$ is governed by the discrete Boltzmann equation with the BGK collision operator:

(2) $$f_{i}\left(x+e_{i}\Delta t,t+\Delta t\right)-f_{i}\left(x,t\right)=-\frac{1}{\tau }\left(f_{i}\left(x,t\right)-f_{i}^{eq}\left(x,t\right)\right),$$

where ${\left\{f_{i}\right\}}_{i=0}^{Q-1}$ are the discrete distributions along lattice velocities $e_{i}$, *Q* represents the total number of discrete velocities, τ is the relaxation time related to viscosity, and $f_{i}^{eq}$ is the equilibrium distribution. By lifting the dynamics into this mesoscopic phase space, LBM captures non-equilibrium kinetic states that store detailed morphological information, thereby enabling the generation of high-fidelity signals without suffering from the over-smoothing characteristic of pure N-S constraints.

#### 2.2.2. Neural Mesoscopic Discretization

**Figure 4:**
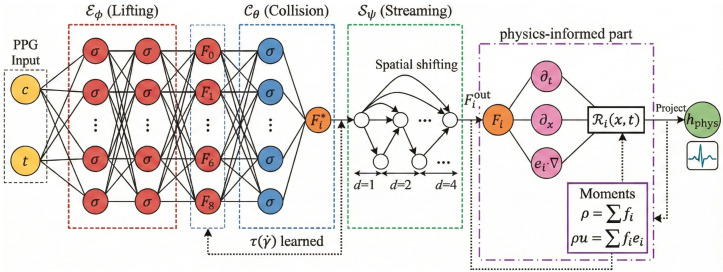
Architecture of the Implicit Lattice Boltzmann Physics Encoder. The macroscopic input *c* (PPG) is lifted by $E_{\phi }$ into mesoscopic distribution functions $F_{i}$. The neural collision operator $C_{\theta }$ learns the dynamic relaxation time τ to output post-collision states $F_{i}^{*}$. The streaming operator $S_{\psi }$ advects these states via spatial shifting (stride *d*) to yield $F_{i}^{out}$. Concurrently, the physics-informed module computes the governing equation residual $R_{i}$ using differential operators ($\partial_{t},\partial_{x}$). Finally, the evolved distributions are projected back to macroscopic moments (ρ,ρu) to form the physical condition embedding $h_{phys}$.

As illustrated in Figure 4, We propose a Neural LBM module that internalizes the physics of Equation (2) into differentiable layers. This module lifts the scalar PPG input into a high-dimensional phase space, evolves it according to kinetic rules, and projects it back to extract hemodynamic invariants.

**Phase Space Lifting (Macroscopic to Mesoscopic).** Treating the 1D temporal signal as our spatial domain, we adopt the classic **D1Q3** discrete velocity configuration (Q=3). The normalized velocity set is $e_{i}\in \left\{-1,0,1\right\}$, representing backward, stationary, and forward wave propagation, respectively.

We define a learnable lifting operator $E_{\phi }:R^{L}\to R^{L\times Q}$ to map the observed scalar PPG signal c(x) to the initial particle distribution functions F(x). This initializes the computational domain by distributing the macroscopic pressure energy into *Q* virtual kinetic modes:

(3) $$F_{i}^{\left(0\right)}\left(x\right)=\mathrm{Softplus}\left(W_{i}c\left(x\right)+b_{i}\right),\;i\in \left\{0,\ldots ,Q-1\right\}.$$

Here, $W_{i}$ and $b_{i}$ denote the unified notation for the learnable weight matrices and bias vectors of the projection layer, respectively. The Softplus activation ensures the non-negativity of the distribution functions, adhering to the physical requirement that particle density cannot be negative.

**Learnable Collision (Rheological Modeling).** The collision term in Equation (2) dictates how distributions relax towards equilibrium, implicitly modeling the fluid’s viscosity. Since real blood exhibits non-Newtonian behavior (shear-thinning) where τ varies dynamically, a fixed collision parameter is insufficient. We implement a *Generalized Neural Collision Operator* ${\tilde{C}}_{\theta }$ using a channel-mixing 1×1 convolution layer:

(4) $${\tilde{C}}_{\theta }{\left(F\left(x,t\right)\right)}_{i}\approx -\frac{1}{\tau (F)}\left(f_{i}-f_{i}^{eq}\right).$$

To strictly conserve mass and momentum, we apply a **Moment Projection** that subtracts the raw mass ($\Delta \rho =\sum_{i}{\tilde{C}}_{\theta ,i}$) and momentum ($\Delta m=\sum_{i}e_{i}{\tilde{C}}_{\theta ,i}$) residuals from the neural output:

(5) $$C_{\theta ,i}={\tilde{C}}_{\theta ,i}-\frac{\Delta \rho }{Q}-\frac{3e_{i}\Delta m}{Q}.$$

The post-collision distribution is then updated as follows:

(6) $$F_{i}^{*}\left(x,t\right)=F_{i}\left(x,t\right)+C_{\theta ,i}.$$

By learning the collision kernel from data under these conservation constraints, this layer adaptively models the complex viscoelastic interactions between the blood and the arterial wall, which are difficult to derive analytically.

**Streaming as Temporal Advection.** The left-hand side of Equation (2) represents the streaming step, describing the exact advection of particles. In our neural formulation, we implement this as a deterministic shift operation. For each latent velocity channel *k*, the features are shifted temporally by a stride $d_{k}$ proportional to the lattice velocity vector $e_{k}$:

(7) $$F_{k}^{out}\left(x\right)=\mathrm{Shift}\left(F_{k}^{*}\left(x\right),\mathrm{step}=d_{k}\right).$$

Unlike standard convolution which mixes local information, this structured shifting strictly enforces the causality of wave propagation, mimicking the physical travel of the pulse wave along the vessel without numerical dissipation.

**Macroscopic Moment Projection.** Finally, we recover the macroscopic hemodynamic features by computing the statistical moments of the evolved distributions, as dictated by Kinetic Theory. The density ρ (zeroth moment) and momentum ρu (first moment) are computed as follows:

(8) $$\rho \left(x\right)=\sum_{i=0}^{Q-1}F_{i}^{out}\left(x\right),\;\rho u\left(x\right)=\sum_{i=0}^{Q-1}F_{i}^{out}\left(x\right)e_{i}.$$

We concatenate these physically derived moments to form the final condition embedding $h_{phys}=\left[\rho ,\rho u\right]$. This provides the diffusion model with rigorous descriptors of the blood flow state—such as local pressure and wall shear stress trends—ensuring the synthesized ECG is physiologically grounded.

### 2.3. Region-Disentangled Diffusion Backbone

#### 2.3.1. Dual-Branch Architecture for Structural-Morphological Synergy

To synthesize high-fidelity ECG signals, the generative model must simultaneously capture fine-grained morphological details and global topological rhythms. Standard U-Net architectures, however, often struggle to balance these conflicting objectives within a single output stream. To address this, we propose a Region-Disentangled U-Net that decouples the reconstruction task into two specialized pathways sharing a common feature extractor.

The backbone network processes the noisy latent state $x_{t}\in R^{C\times L}$, combined with the timestep embedding τ and the physical condition $h_{phys}$. A shared encoder first extracts a multi-scale latent representation $h_{dec}$. Subsequently, the architecture bifurcates into a Geometry Branch and a Topology Branch. The *Geometry Branch* focuses on local signal reconstruction by predicting the noise residual $\epsilon_{\theta }$. It projects the latent features back to the signal space via a convolutional head:

(9) $$\epsilon_{\theta }\left(x_{t},t,h_{phys}\right)=W_{noise}*\mathrm{SiLU}\left(\mathrm{GN}\left(h_{dec}\right)\right)+b_{noise}\in R^{L},$$

where ∗ denotes the convolution operation, and GN represents Group Normalization. This output is primarily responsible for recovering the high-frequency waveform details required for the reverse diffusion process.

Simultaneously, the *Topology Branch* functions as an auxiliary semantic segmentation module. Instead of predicting signal values, it estimates a probability mask $\hat{r}\in {\left[0,1\right]}^{L}$ that highlights the Regions of Interest (ROI), specifically the QRS complexes. This is achieved through a separate projection head followed by a sigmoid activation:

(10) $$\hat{r}=\sigma \left(W_{reg}*h_{dec}+b_{reg}\right).$$

By jointly optimizing this branch, we enforce a strong inductive bias that compels the shared encoder to learn representations that are not only effective for denoising but also semantically aware of the underlying cardiac cycle phases, ensuring global rhythmic coherence.

#### 2.3.2. Physically Guided Cross-Attention Mechanism

To rigorously condition the generative process on hemodynamic laws, we introduce a Physically Guided Cross-Attention mechanism embedded at the bottleneck of the U-Net. This layer acts as a dynamic interface, aligning the fluid domain features extracted by the LBM encoder with the electrical domain latents of the diffusion model.

Let $Z\in R^{L^{\prime }\times D_{z}}$ denote the intermediate feature map of the noisy ECG in the U-Net bottleneck, and $h_{phys}\in R^{L^{\prime }\times D_{p}}$ denote the downsampled hemodynamic features. We formulate the interaction by projecting these inputs into query, key, and value subspaces:

(11) $$Q=ZW_{Q},\;K=h_{phys}W_{K},\;V=h_{phys}W_{V},$$

where $W_{Q}\in R^{D_{z}\times d_{k}}$, and $W_{K},W_{V}\in R^{D_{p}\times d_{k}}$ are learnable projection matrices. The attention-driven feature injection is then computed as follows:

(12) $$Z^{\prime }=Z+\mathrm{Softmax}\left(\frac{QK^{T}}{\sqrt{d_{k}}}\right)V.$$

**Figure 5:**
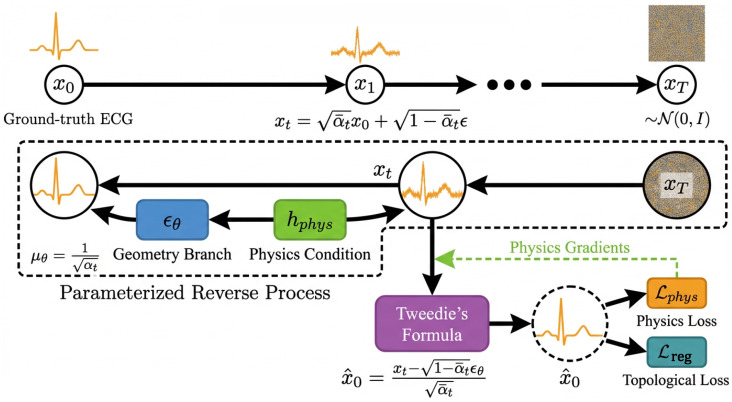
Physics-Informed Conditional Diffusion Framework.

In this formulation, the attention matrix $M=\mathrm{Softmax}\left(QK^{T}/\sqrt{d_{k}}\right)\in R^{L^{\prime }\times L^{\prime }}$ effectively models the physiological transfer function between the two modalities. It computes the temporal correlation between specific hemodynamic events and electrical triggers. Unlike simple concatenation, this mechanism allows the model to adaptively compensate for the Pulse Transit Time the variable delay between electrical activation and mechanical pulse—thereby ensuring precise temporal synchronization in the reconstructed signal (Figure 5).

### 2.4. Physics-Informed Generative Learning

#### 2.4.1. Conditional Diffusion Dynamics

We formulate the ECG synthesis as a conditional diffusion process involving a forward trajectory that corrupts the data and a learnable reverse trajectory that reconstructs it. The forward diffusion process is defined as a fixed Markov chain that gradually adds Gaussian noise to the ground-truth signal $x_{0}$ according to a variance schedule ${\left\{\beta_{t}\in \left(0,1\right)\right\}}_{t=1}^{T}$. For each step *t*, the transition probability is given by $q\left(x_{t}|x_{t-1}\right)=N\left(x_{t};\sqrt{1-\beta_{t}}x_{t-1},\beta_{t}I\right)$. By defining $\alpha_{t}=1-\beta_{t}$ and ${\bar{\alpha }}_{t}=\prod_{s=1}^{t}\alpha_{s}$, we can derive the marginal distribution of $x_{t}$ conditioned on $x_{0}$ in closed form. This allows us to sample $x_{t}$ at any arbitrary timestep directly using the reparameterization trick:

(13) $$x_{t}=\sqrt{{\bar{\alpha }}_{t}}x_{0}+\sqrt{1-{\bar{\alpha }}_{t}}\epsilon ,\;\mathrm{where}\;\epsilon ∼N\left(0,I\right).$$

The generative reverse process aims to invert this diffusion chain to recover $x_{0}$ from a standard Gaussian prior $x_{T}∼N\left(0,I\right)$, explicitly conditioned on the hemodynamic features $h_{phys}$. Since the exact posterior $q(x_{t-1}|x_{t})$ is intractable, we approximate it with a parameterized Gaussian distribution

(14) $$p_{\theta }\left(x_{t-1}|x_{t},h_{phys}\right)=N\left(x_{t-1};\mu_{\theta }\left(x_{t},t,h_{phys}\right),\sigma_{t}^{2}I\right)$$

The variance $\sigma_{t}^{2}$ is typically fixed to $\beta_{t}$ or ${\tilde{\beta }}_{t}=\frac{1-{\bar{\alpha }}_{t-1}}{1-{\bar{\alpha }}_{t}}\beta_{t}$. The core learning objective lies in estimating the mean $\mu_{\theta }$. By matching the generative distribution to the true posterior $q(x_{t-1}|x_{t},x_{0})$, the optimal mean can be parameterized as a linear combination of the current noisy state and a predicted noise component:

(15) $$\mu_{\theta }\left(x_{t},t,h_{phys}\right)=\frac{1}{\sqrt{\alpha_{t}}}\left(x_{t}-\frac{\beta_{t}}{\sqrt{1-{\bar{\alpha }}_{t}}}\epsilon_{\theta }\left(x_{t},t,h_{phys}\right)\right).$$

Here, $\epsilon_{\theta }$ is the function approximated by our Geometry Branch, which learns to predict the noise ϵ present in $x_{t}$ given the physical condition.

#### 2.4.2. Physics-Consistent Estimation via Tweedie’s Formula

A fundamental challenge in incorporating physical laws into diffusion models is that constraints such as signal continuity and derivatives are defined on the clean signal manifold $x_{0}$, whereas the network operates on the noisy latent space $x_{t}$. Applying physical regularization directly to $x_{t}$ is ineffective due to the dominance of high-frequency Gaussian noise. To resolve this, we employ Tweedie’s formula to analytically estimate the denoised signal ${\hat{x}}_{0}$ from the current noisy state $x_{t}$ and the predicted noise $\epsilon_{\theta }$. By rearranging the forward marginal equation, we obtain the projection operator:

(16) $${\hat{x}}_{0}\left(x_{t},\epsilon_{\theta }\right)=\frac{x_{t}-\sqrt{1-{\bar{\alpha }}_{t}}\epsilon_{\theta }\left(x_{t},t,h_{phys}\right)}{\sqrt{{\bar{\alpha }}_{t}}}.$$

This formulation acts as a single-step denoising approximation. It enables us to enforce the physics-based loss $L_{phys}$ directly on the estimated clean manifold ${\hat{x}}_{0}$ at every training step *t*, ensuring that the gradients backpropagated to the network effectively guide the generation towards hemodynamically consistent waveforms.

#### 2.4.3. Multi-Objective Optimization

The training of PhysDiff-LBM is governed by a composite objective function designed to jointly satisfy signal fidelity, structural alignment, and physical consistency requirements. The total loss $L_{total}$ is constructed as a weighted sum of three complementary terms:

(17) $$L_{total}=\lambda_{diff}L_{diff}+\lambda_{reg}L_{reg}+\lambda_{phys}L_{phys},$$

where $\lambda_{diff},\lambda_{reg},\lambda_{phys}$ are hyperparameters balancing the optimization landscape.

The primary reconstruction capability is driven by the denoising loss $L_{diff}$, which minimizes the variational upper bound on the negative log-likelihood. Following the reparameterization of the reverse process mean, this simplifies to minimizing the $L_{2}$ distance between the sampled Gaussian noise ϵ and the noise residual predicted by the network:

(18) $$L_{diff}=E_{t,x_{0},\epsilon }\left[∥\epsilon -\epsilon_{\theta }\left(x_{t},t,h_{phys}\right){∥}_{2}^{2}\right].$$

While this term ensures pixel-level accuracy, standard MSE optimization often prioritizes local smoothness at the expense of global structural coherence. To mitigate this, we simultaneously optimize a topology loss $L_{reg}$ through the auxiliary Topology Branch. By employing a Binary Cross-Entropy objective against the ground-truth QRS mask *r*, formulated as $L_{reg}=\mathrm{BCE}\left(r,\hat{r}\right)$, we compel the shared encoder to explicitly capture the cardiac rhythm, ensuring the generated waveforms are topologically aligned with the physiological cycle.

Crucially, to ensure the synthesis adheres to fluid dynamic laws, we incorporate a hemodynamic consistency loss $L_{phys}$. Leveraging the Tweedie’s projection derived in Equation (16), we explicitly penalize discrepancies between the electrical evolution of the estimated signal and the fluid momentum extracted by the LBM encoder:

(19) $$L_{phys}=E_{t,x_{0},\epsilon }\left[{∥\frac{\partial {\hat{x}}_{0}}{\partial \tau }-P_{\phi }\left(h_{phys}\right)∥}_{2}^{2}\right].$$

Here, $\frac{\partial }{\partial \tau }$ represents the temporal derivative operator. This term enforces the constraint that the gradient of the generated ECG must correspond to the hemodynamic momentum flux $P_{\phi }\left(h_{phys}\right)$, thereby effectively suppressing non-physiological hallucinations and ensuring strict cross-domain physical coupling.

## 3. Experiments

### 3.1. Experimental Setup

#### 3.1.1. Datasets and Protocol

We evaluate PhysDiff-LBM using four datasets divided into reconstruction benchmarks:

**Training and Reconstruction Benchmarks**:

- **MIMIC-III Waveform Database**: A large-scale ICU dataset from which we curated 5000+ subjects. It represents complex, noisy hemodynamic conditions, serving as the primary training corpus [3,28].
- **VitalDB**: High-fidelity intraoperative recordings capturing hemodynamics under anesthesia. These dataset is used to assess model generalization across distinct physiological states outside the ICU domain [29].
- **MIMIC PERform AF Dataset**: This binary classification task focuses on distinguishing Atrial Fibrillation from Normal Sinus Rhythm. Since AF is characterized by the absence of P-waves and irregular R-R intervals, this task explicitly tests the model’s ability to capture high-frequency atrial depolarization details and temporal rhythm consistency [30,31].
- **PhysioNet Challenge 2015 Dataset**: We performed a 5-class arrhythmia classification task covering Asystole, Bradycardia, Tachycardia, Ventricular Tachycardia (VTA), and Ventricular Fibrillation (VFB). This challenging scenario requires the reconstructed signals to reflect diverse morphological anomalies, ranging from extreme rate variations to complete waveform disorganization [32].

#### 3.1.2. Preprocessing

All signals were resampled to 128 Hz and segmented into non-overlapping 4-s windows (L=512). We utilized *NeuroKit2* for signal conditioning, applying standard bandpass filtering for PPG and the Pan-Tompkins algorithm for ECG cleaning to remove artifacts. To stabilize the diffusion process, we applied instance-wise Min-Max normalization to scale each window *x* to the range [−1,1]:

(20) $$\tilde{x}=2\cdot \frac{x-\min(x)}{\max(x)-\min(x)}-1.$$

Furthermore, to supervise the topological branch, we generated binary Region-of-Interest (ROI) masks $r\in {\left\{0,\;1\right\}}^{L}$. Using R-peaks detected via the Pan-Tompkins method, we defined the QRS complex region as a 32-sample window centered on each peak index $t_{p}$:

(21) $$r_{t}=I(|t-t_{p}\left|\;\leq \;16\right),$$

where I(·) is the indicator function, effectively highlighting the structural cardiac events.

#### 3.1.3. Baselines

We compare PhysDiff-LBM against three representative state-of-the-art approaches:

- **CardioGAN**: An attention-based SOTA GAN framework utilizing dual discriminators for ECG synthesis. It serves as the primary adversarial baseline to evaluate the trade-off between perceptual quality and training stability [33].
- **PhysDiff-NS**: A PINN-based variant of our model where the LBM encoder is replaced by explicit 1D Navier–Stokes residual regularization. This baseline is designed to benchmark our implicit kinetic formulation against traditional macroscopic fluid constraints with constant viscosity.
- **RecQSR**: A specialized deterministic framework focusing on fine-grained QRS complex reconstruction. It represents the state-of-the-art in non-generative, regression-based deep learning approaches [34].

#### 3.1.4. Implementation Details and Environment

To ensure the reproducibility of our study, we detail the computational environment and software frameworks utilized for model development and evaluation. All neural network architectures, including the implicit LBM encoder and the region-disentangled diffusion backbone, were implemented using the **PyTorch** deep learning framework (version 2.6.0) in Python 3.10. For physiological signal preprocessing, filtering, and R-peak detection, we leveraged the NeuroKit2 and SciPy libraries. All model training, hyperparameter tuning, and performance evaluations were accelerated using CUDA 11.7 and conducted on a high-performance Linux workstation equipped with a single NVIDIA GeForce RTX 4090 GPU (NVIDIA Corporation, Santa Clara, CA, USA, 24 GB VRAM).

### 3.2. Comparative Experiment

#### 3.2.1. Evaluation Metrics

To assess the performance of PhysDiff-LBM from morphological, clinical, and distributional perspectives, we employ three key metrics:

- **Waveform Fidelity (RMSE)**: Measures point-wise reconstruction accuracy. $\mathrm{RMSE}=\sqrt{\frac{1}{L}\sum {\left(x_{t}-{\hat{x}}_{t}\right)}^{2}}$.
- **Clinical Accuracy (HR-MAE)**: Evaluates diagnostic validity by calculating the absolute deviation in Heart Rate (BPM) derived via the Pan-Tompkins algorithm: $\mathrm{HR}-\mathrm{MAE}=\frac{1}{N}\sum \left|{\mathrm{BPM}}_{gt}-{\mathrm{BPM}}_{pred}\right|$.
- **Distributional Consistency (FD)**: Fréchet Distance measures the Wasserstein-2 distance between the empirical distributions of the raw generated and real ECG signals, treating each one-dimensional signal window directly as a high-dimensional vector, to assess structural realism and mode coverage.

#### 3.2.2. Results

**Figure 6:**
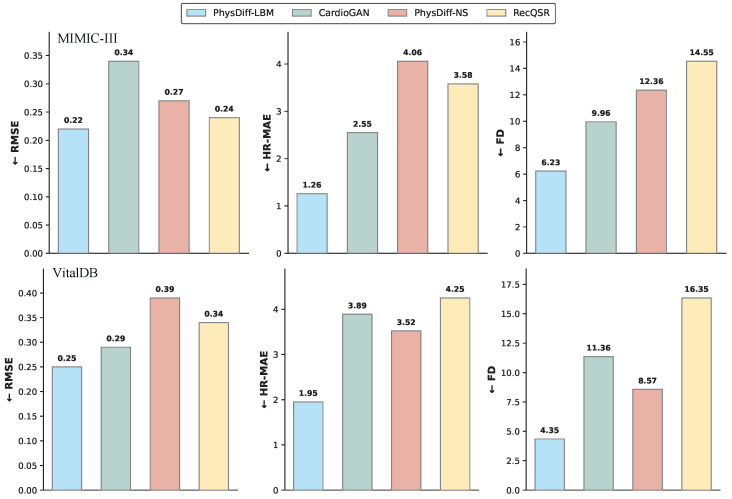
Quantitative comparison of reconstruction performance across MIMIC-III and VitalDB datasets. Lower values indicate better performance for all metrics (↓).

**Figure 7:**
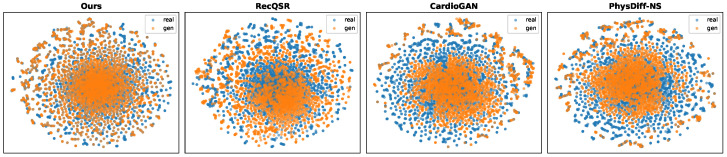
t-SNE visualization of feature embeddings for real (blue) and generated (orange) ECG signals across different methods. Closer overlap indicates better distributional alignment.

**Figure 8:**
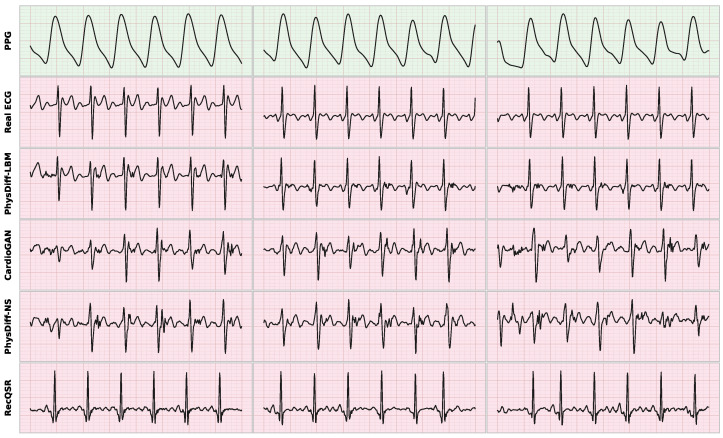
Qualitative comparison of ECG waveforms reconstructed from PPG signals across different methods. From top to bottom: input PPG, ground truth ECG, and outputs from PhysDiff-LBM (ours), CardioGAN, PhysDiff-NS, and RecQSR.

We evaluate the complete methods on the two reconstruction benchmarks—MIMIC-III Waveform Database and VitalDB—using the three aforementioned metrics, with quantitative results summarized in Figure 6, Figure 7 and Figure 8.

#### 3.2.3. Overall Performance Analysis

As illustrated in Figure 6, PhysDiff-LBM consistently achieves superior performance across all evaluation metrics on both benchmarks. The improvements are particularly pronounced in distributional consistency and clinical accuracy, indicating that our physics-constrained framework not only reconstructs morphologically accurate waveforms but also preserves the underlying physiological semantics essential for clinical interpretation. CardioGAN exhibits the weakest performance among all compared methods, particularly in terms of distributional alignment. This degradation can be attributed to the inherent mode collapse and training instability issues associated with adversarial training paradigms, while GANs excel at generating perceptually plausible samples, they often fail to capture the full diversity of cardiac morphologies, leading to hallucinated features that deviate from physiologically valid patterns. PhysDiff-NS, which employs explicit Navier–Stokes residual constraints, demonstrates competitive reconstruction fidelity but falls short of PhysDiff-LBM in capturing fine-grained morphological details. This performance gap highlights the limitations of macroscopic fluid equations with constant viscosity assumptions, which cannot adequately model the spatially heterogeneous and nonlinear dynamics of cardiovascular systems. RecQSR, as a regression-based deterministic approach, achieves reasonable waveform fidelity but exhibits limited capacity in preserving distributional characteristics, tending to produce over-smoothed outputs that average out subtle morphological variations.

The representative waveform comparison in Figure 8 provides intuitive evidence of the qualitative differences among methods. PhysDiff-LBM faithfully reconstructs the characteristic morphological features including sharp R-peaks, appropriate QRS complex duration, and physiologically consistent ST-T wave transitions. In contrast, baseline methods exhibit various artifacts: CardioGAN produces irregular baseline wandering and spurious oscillations; PhysDiff-NS generates overly smooth transitions that blur the boundaries between waveform components; RecQSR tends to miss subtle inflection points in the P-wave and T-wave regions. Furthermore, the t-SNE projections in Figure 7 reveal distinct clustering behaviors across methods. PhysDiff-LBM achieves the highest degree of overlap between real and generated distributions, indicating that our model successfully captures the intrinsic manifold structure of ECG signals. The compact and well-aligned clusters suggest that the LBM-encoded physics constraints effectively regularize the generation process, preventing the model from drifting into physiologically implausible regions of the feature space.

Notably, PhysDiff-LBM maintains robust performance on the out-of-distribution VitalDB dataset, which captures hemodynamics under anesthesia-induced physiological perturbations, while all methods exhibit some degree of performance degradation when transferring from ICU to intraoperative settings, our approach demonstrates the smallest generalization gap. This robustness stems from the physics-informed inductive bias embedded in our LBM encoder, which captures domain-invariant hemodynamic principles rather than dataset-specific statistical correlations. In contrast, our LBM-based formulation operates at the mesoscopic scale, enabling adaptive viscosity encoding through learned collision operators that better reflect the complex rheological properties of blood flow, thereby achieving superior cross-domain transferability and clinical reliability.

### 3.3. Interpretability and Mesoscopic Physics Analysis

**Figure 9:**
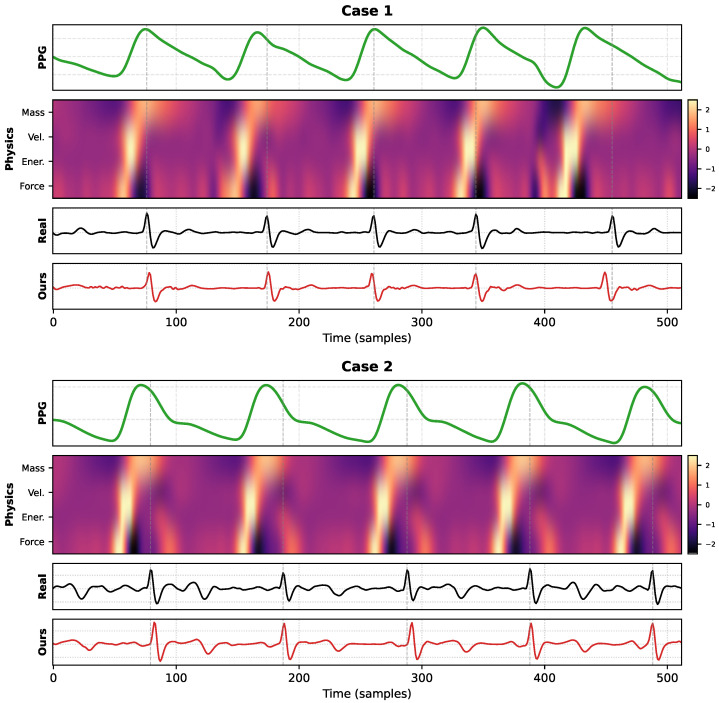
Visualization of the physics-guided ECG inference process.

To investigate the underlying mechanism of PhysDiff-LBM, we visualize the inference dynamics for many representative samples in Figure 9. This qualitative breakdown reveals how the mesoscopic constraints bridge the domain gap between hemodynamic boundary conditions, represented by the PPG, and the electrophysiological responses of the ECG.

The visualization of the latent manifold, depicted in the second row of Figure 9, provides empirical evidence that our model learns explicit fluid dynamic descriptors rather than abstract statistical features. The activation patterns in the “Velocity” and “Kinetic Energy” channels are not random but exhibit high-intensity responses corresponding precisely to the maximum systolic upstroke of the input PPG. From a fluid mechanics perspective, these activations represent the gradient of blood volume changes, denoted as ∂P/∂t, and the associated momentum flux. Crucially, observing the alignment with the gray dashed lines reveals a consistent physiological phenomenon where the peak activation of these mesoscopic physical features lags slightly behind the electrical R-peaks. This latency corresponds to the Pulse Transit Time, or PTT, which is the interval required for the pressure wave generated by ventricular contraction to propagate to the peripheral measurement site.

This observation suggests that PhysDiff-LBM has successfully modeled the *inverse* hemodynamic transfer function. Instead of simply translating waveform textures, the diffusion process is guided by the collision operator to locate the precise moment of maximum momentum generation associated with cardiac ejection, effectively back-tracing the preceding electrical depolarization event. This physics-based reasoning explains the superior temporal precision of the model observed in quantitative results. Even in the presence of irregular heartbeats or baseline wander, the rigid coupling between mechanical flow captured by LBM and the electrical trigger of the ECG forces the generated QRS complexes to align strictly with the ground truth. This mechanism effectively prevents the phase shifts commonly seen in pure end-to-end regression baselines.

Furthermore, the “Force” channel in the heatmap acts as a second-order regularization term. By highlighting regions of high acceleration, defined as rapid changes in flow velocity, it enforces structural sharpness in the reconstructed signal. This is evident in the reconstructed ECG shown in the bottom row, where the QRS complexes maintain high-frequency fidelity without the over-smoothing artifacts typical of MSE-based approaches. Consequently, the LBM module acts as a dynamic filter that allows the diffusion model to distinguish between genuine high-frequency cardiac features possessing corresponding hemodynamic acceleration signatures and random Gaussian noise lacking physical momentum support, thereby ensuring robustness across diverse physiological states.

### 3.4. Ablation Study

To systematically evaluate the contribution of each proposed component, we conduct comprehensive ablation experiments on both MIMIC-III and VitalDB benchmarks. We design four ablation variants by progressively removing or replacing key modules from the full PhysDiff-LBM framework, as summarized in Table 1.

The first variant (M1) replaces the entire implicit LBM physics encoding module with a standard 1D convolutional encoder, assessing the contribution of mesoscopic physical modeling compared to purely data-driven feature extraction. The second variant (M2) substitutes the dilated convolutions in the streaming operator with standard convolutions of unit dilation rate, examining the role of multi-scale feature propagation in preserving pulse wave phase information. The third variant (M3) eliminates the region detection head $H_{\mathrm{region}}$ along with the auxiliary segmentation objective $L_{\mathrm{reg}}$, investigating the contribution of explicit cardiac rhythm supervision. The fourth variant (M4) replaces the physically guided cross-attention mechanism with simple channel-wise concatenation, evaluating the necessity of dynamic temporal alignment for Pulse Transit Time compensation.

Table 2 presents the quantitative performance of all ablation variants across both benchmarks. The complete PhysDiff-LBM framework (M0) consistently achieves the best performance across all metrics, validating the effectiveness of our integrated design.

The ablation results reveal several important observations regarding the contribution of each component. Most notably, the removal of the LBM encoder (M1) leads to catastrophic performance degradation across all metrics. This substantial gap confirms that the mesoscopic physics-informed encoding constitutes the core contribution of our framework. Without the implicit hemodynamic constraints embedded in the LBM formulation, the model degenerates into a purely data-driven paradigm that fails to capture the underlying fluid dynamic principles governing cardiovascular wave propagation.

Interestingly, we observe distinct degradation patterns across different ablation variants, suggesting that each component addresses complementary aspects of the reconstruction problem. The removal of dilated streaming (M2) causes the most severe degradation in FD, while maintaining relatively modest HR-MAE increase. This asymmetric pattern indicates that multi-scale feature propagation primarily affects the global distributional consistency of generated waveforms rather than local peak detection accuracy. The dilated convolutions enable the model to capture long-range temporal dependencies that are essential for preserving the characteristic shape of the entire cardiac cycle.

In contrast, the topology branch removal (M3) exhibits an opposite degradation pattern, with HR-MAE surging dramatically while FD remains relatively controlled. This observation validates our hypothesis that explicit cardiac rhythm supervision through the auxiliary segmentation task provides crucial inductive bias for learning the temporal structure of ECG signals. Without direct guidance on QRS complex localization, the model loses awareness of the underlying topological organization, resulting in rhythmic hallucinations where cardiac cycles may exhibit plausible morphology but incorrect temporal placement.

The cross-attention mechanism (M4) demonstrates the most balanced contribution across metrics, with moderate degradation in both HR-MAE and FD. This suggests that the dynamic temporal alignment provided by attention weights benefits both local and global reconstruction quality by adaptively compensating for the variable Pulse Transit Time between cardiac electrical activation and peripheral pulse arrival.

Across both datasets, the ranking of variants remains largely consistent, with M1 performing worst, followed by M2 and M3 with complementary weakness profiles, and M4 exhibiting the smallest overall degradation. The consistent performance gap between M0 and all ablation variants validates that all proposed components contribute synergistically to the final reconstruction quality, with the LBM physics encoding serving as the foundational element upon which other components build.

### 3.5. Downstream Clinical Validation

Beyond morphological reconstruction fidelity, the ultimate criterion for the generated ECGs is their clinical utility—specifically, whether they preserve the pathological biomarkers required for automated diagnosis. To evaluate this, we trained a standard VGG-19 [35,36] classifier on ground-truth real ECG signals to serve as a fixed diagnostic evaluator. This pre-trained network was then used to predict cardiac pathologies from three types of input signals: the ground-truth Real ECG, the reconstructed ECG generated by each method (Gen ECG), and the raw PPG signal. High classification performance on generated ECGs indicates that the generative model successfully recovers the subtle diagnostic features rather than merely fitting low-frequency trends.

We conducted evaluations on two distinct diagnostic tasks (Table 3):

#### 3.5.1. Evaluation Metrics

To comprehensively assess the diagnostic fidelity of reconstructed ECG signals, we employ a suite of classification metrics that capture both overall performance and class-specific diagnostic accuracy. Let TP, TN, FP, and FN denote the number of true positives, true negatives, false positives, and false negatives, respectively.

Accuracy represents the proportion of correctly classified samples among all predictions, providing an overall measure of classification correctness:

(22) $$\mathrm{Accuracy}=\frac{TP+TN}{TP+TN+FP+FN}$$

Precision is defined as the ratio of true positive predictions to all positive predictions, indicating the reliability of positive classifications:

(23) $$\mathrm{Precision}=\frac{TP}{TP+FP}$$

Recall, also known as sensitivity, represents the ratio of true positive predictions to all actual positive samples, reflecting the model’s ability to detect pathological conditions:

(24) $$\mathrm{Recall}=\frac{TP}{TP+FN}$$

The F1-Score is the harmonic mean of precision and recall, providing a more balanced performance measure when class distributions are imbalanced:

(25) $$F1=2\times \frac{\mathrm{Precision}\times \mathrm{Recall}}{\mathrm{Precision}+\mathrm{Recall}}=\frac{2TP}{2TP+FP+FN}$$

The Area Under the ROC Curve (AUC) is a threshold-independent metric that quantifies the discriminative capability across all classification thresholds by computing the area under the Receiver Operating Characteristic curve. AUC values range from [0, 1], where 0.5 indicates random guessing and 1.0 represents perfect classification [37].

For the multi-class arrhythmia diagnosis task, we extend these metrics using macro-averaging. Let *K* denote the total number of classes, and let ${\mathrm{Precision}}_{k}$ and ${\mathrm{Recall}}_{k}$ represent the precision and recall for class *k*, respectively. The macro-averaged precision and recall are defined as:

(26) $${\mathrm{Precision}}_{\mathrm{macro}}=\frac{1}{K}\sum_{k=1}^{K}{\mathrm{Precision}}_{k},\;{\mathrm{Recall}}_{\mathrm{macro}}=\frac{1}{K}\sum_{k=1}^{K}{\mathrm{Recall}}_{k}$$

Macro-averaging ensures equal weighting across all five arrhythmia categories, preventing dominant classes from masking performance on rare but clinically critical conditions such as Ventricular Fibrillation.

To establish a comprehensive benchmark, we evaluate and compare three types of signals: (1) Real ECG, the ground-truth ECG recordings serving as the upper-bound reference; (2) Gen ECG, the ECG waveforms reconstructed by PhysDiff-LBM and baseline methods; and (3) PPG, the raw photoplethysmography input signal serving as the lower-bound reference. The diagnostic gap between Gen ECG and Real ECG quantifies the information loss during reconstruction, while the improvement from PPG to Gen ECG demonstrates the value added by the translation process. A successful reconstruction method should achieve Gen ECG performance approaching that of Real ECG while substantially exceeding PPG performance.

#### 3.5.2. Clinical Validation on Atrial Fibrillation

**Figure 10:**
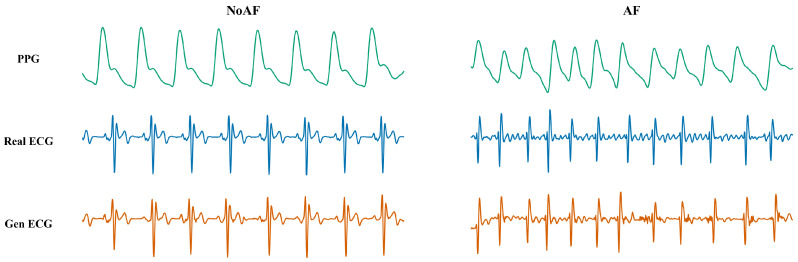
Representative PPG, Real ECG, and Gen ECG signals for NoAF and AF.

Before quantifying the diagnostic performance, we first visually inspect the reconstruction quality across different cardiac rhythms. Figure 10 illustrates the waveforms of Raw PPG, Real ECG, and our generated Gen ECG for both Normal Sinus Rhythm (NoAF) and Atrial Fibrillation (AF). As observed, the Raw PPG signals (top row) exhibit smoothed peaks, where the fine-grained timing of depolarization is often obscured. In contrast, the Gen ECG (bottom row) sharpens these features, strictly aligning with the Real ECG (middle row). Crucially, in the AF scenario, our model captures the characteristic irregularity of the rhythm, a biomarker essential for clinical diagnosis that is less distinct in the PPG domain.

**Figure 11:**
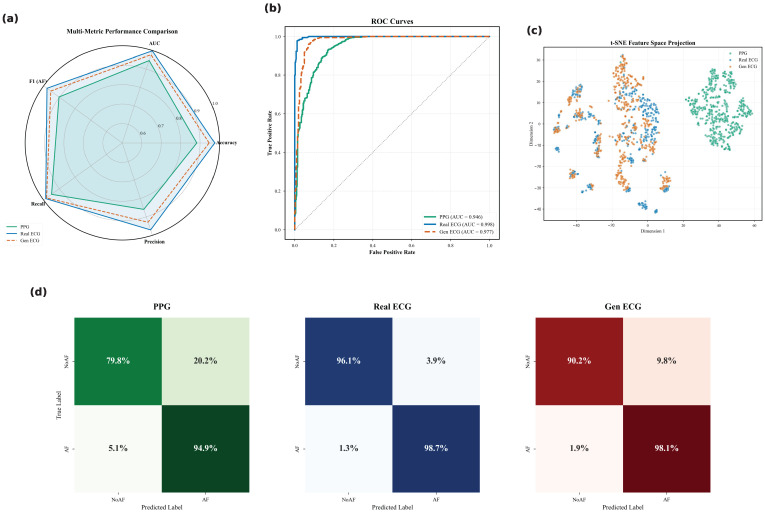
Detailed diagnostic performance analysis on the test set. (**a**–**c**) Confusion Matrices for Raw PPG, Gen ECG, and Real ECG, respectively, illustrating the distribution of true positives and misclassification types. (**d**) ROC Curves comparing the sensitivity and specificity trade-offs across the three modalities. The Gen ECG (**b**) shows a diagonal dominance comparable to the Real ECG (**c**), significantly reducing the off-diagonal errors observed in PPG (**a**).

To validate the clinical utility, we utilized the pre-trained VGG-19 network to classify cardiac pathologies. Table 4 summarizes the performance metrics with 95% confidence intervals (CIs), while Figure 11 provides a detailed visualization of the classification boundaries and error distribution.

We computed 95% CIs via 1000-resample bootstrapping. Statistical tests (paired permutation for F1 and DeLong’s for AUC) confirm that our Gen ECG significantly outperforms the Raw PPG baseline (p<0.001).

The quantitative results reveal distinct performance characteristics inherent to each signal modality. The Raw PPG baseline demonstrates a notable imbalance between sensitivity (Recall) and reliability (Precision), while the optical signal successfully captures the majority of pathological events, its comparatively lower Precision suggests a tendency toward false positives. This limitation likely stems from the susceptibility of PPG to motion artifacts, which can mimic the irregularities of arrhythmia.

The transition from PPG to Gen ECG addresses this critical bottleneck. As visualized in the confusion matrices in Figure 11, the Gen ECG significantly tightens the classification distribution. Comparing the off-diagonal elements between Figure 11a,b, we observe that the generative process effectively suppresses the ambiguity that leads to misclassification in the PPG domain. By enforcing electrophysiological constraints, the model acts as a semantic filter, reducing the noise that classifiers typically confound with pathological features.

Furthermore, the performance of the generated signals closely converges with that of the ground-truth Real ECG. The minimal gap across all metrics (and overlapping confidence intervals in certain metrics) indicates that the information loss during the cross-domain translation is negligible for diagnostic purposes. The high alignment in both the statistical metrics (Table 4) and the ROC space (Figure 11d) confirms that the reconstructed ECGs retain the subtle, clinically relevant biomarkers necessary for automated decision-making.

#### 3.5.3. Clinical Validation on Multi-Class Arrhythmia Diagnosis

Beyond binary classification, we further evaluate the diagnostic fidelity of generated ECGs on a more challenging multi-class arrhythmia task using the PhysioNet Challenge 2015 dataset. This benchmark encompasses five distinct cardiac conditions: Asystole, Bradycardia, Tachycardia, Ventricular Tachycardia (VT), and Ventricular Fibrillation (VF/VFib), each presenting unique electrophysiological signatures that must be preserved through the cross-domain translation process.

**Figure 12:**
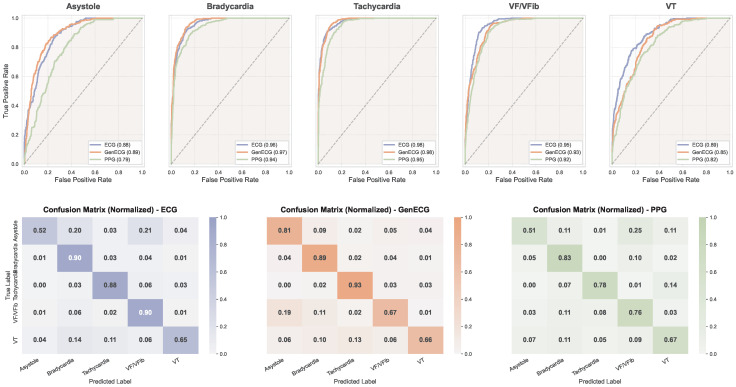
Comprehensive diagnostic performance analysis for multi-class arrhythmia classification. Top row: Per-class ROC curves comparing the discriminative capability of Real ECG, Gen ECG, and Raw PPG across all five arrhythmia categories. Bottom row: Normalized confusion matrices for ECG, Gen ECG, and PPG, illustrating the distribution of correct classifications and inter-class confusion patterns.

Table 5 summarizes the per-class and macro-averaged classification metrics across the three signal modalities. To maintain clarity while ensuring statistical rigor, we report the 95% confidence intervals for the overall Macro Average metrics. Figure 12 provides the corresponding ROC curves and normalized confusion matrices for detailed performance visualization.

The multi-class classification results further substantiate the clinical utility of our generated ECGs. Across all arrhythmia categories, the Gen ECG consistently outperforms the Raw PPG baseline in terms of macro-averaged metrics, with particularly pronounced improvements observed for life-threatening conditions such as Asystole and Ventricular Tachycardia. This enhanced sensitivity for critical cardiac events underscores the capacity of the generative model to recover diagnostically relevant features that are otherwise attenuated in the optical domain.

Notably, the Gen ECG achieves performance that not only approaches but in certain cases surpasses that of the Real ECG reference, especially for rate-related arrhythmias including Bradycardia and Tachycardia. This counterintuitive observation suggests that the generative process may function as an implicit denoising mechanism, yielding cleaner electrophysiological representations that enhance classifier discriminability for conditions primarily characterized by rhythm alterations rather than subtle morphological abnormalities.

The confusion matrices in Figure 12 exhibits considerable inter-class confusion, particularly between conditions sharing similar heart rate profiles but differing in their underlying electrical substrates—such as Asystole versus VF/VFib, and Bradycardia versus VT. These misclassifications are physiologically expected, as the optical signal inherently lacks the resolution to capture morphological distinctions encoded in the electrical domain. By recovering the QRS complex morphology and rhythm regularity, the Gen ECG effectively disambiguates these clinically distinct conditions, achieving diagonal dominance comparable to the Real ECG reference.

The ROC curves further demonstrate that the Gen ECG maintains robust discriminative performance across varying classification thresholds. The curves for Gen ECG closely track those of the Real ECG across most arrhythmia categories while consistently dominating the PPG curves, confirming that the reconstructed signals preserve the essential pathological biomarkers required for reliable automated diagnosis. The marginal performance gap observed in complex conditions such as Ventricular Fibrillation likely reflects the inherent challenge of reconstructing high-frequency chaotic waveforms from bandwidth-limited PPG signals, representing a potential direction for future methodological refinement.

## 4. Discussion

The reconstruction of high-fidelity ECG signals from photoplethysmography represents a fundamental challenge in ubiquitous health monitoring, primarily due to the complex non-linear mapping between the mechanical pulsatile flow and the underlying electrical cardiac activity. Our results demonstrate that PhysDiff-LBM effectively addresses this ill-posed inverse problem by integrating mesoscopic fluid dynamics into a generative diffusion framework. Unlike traditional deep learning approaches that rely solely on statistical correlations, our method leverages the Lattice Boltzmann Method to impose explicit hemodynamic constraints, effectively grounding the generative process in physical reality. This structural coupling ensures that the reconstructed waveforms are not only texturally realistic but also physiologically compliant with the input boundary conditions.

A comparative analysis with state-of-the-art baselines elucidates the specific advantages of our mesoscopic formulation, while adversarial frameworks like CardioGAN excel at generating sharp waveforms, they frequently suffer from mode collapse and “rhythmic hallucination,” where the generated beats fail to align temporally with the input PPG during irregular rhythms. In contrast, PhysDiff-LBM utilizes the kinetic energy and momentum features derived from the PPG to rigidly anchor the temporal alignment, preventing phase shifts even in the presence of arrhythmias. Furthermore, compared to deterministic regression models (e.g., RecQSR) which tend to produce over-smoothed signals by averaging out high-frequency details, our diffusion-based approach preserves the spectral richness of the ECG, capturing subtle morphological variations in the QRS complex and T-wave. Crucially, our method also outperforms macroscopic physics-informed baselines (PhysDiff-NS). We attribute this to the fact that standard Navier–Stokes formulations often assume constant viscosity, which oversimplifies the complex, non-Newtonian behavior of blood flow in microvascular beds. By operating at the mesoscopic scale with a learnable collision operator, PhysDiff-LBM better captures these heterogeneous rheological properties, leading to superior reconstruction fidelity.

The clinical significance of these findings is underscored by the model’s performance in downstream diagnostic tasks. The substantial improvement in Atrial Fibrillation detection and multi-class arrhythmia classification indicates that the generated signals retain the critical diagnostic features necessary for automated screening. By accurately recovering the “irregularly irregular” rhythm characteristic of AF without introducing generation artifacts, PhysDiff-LBM effectively functions as a software-defined sensor enhancement, potentially upgrading standard consumer wearables into medical-grade diagnostic tools. Moreover, the robustness demonstrated on the out-of-distribution VitalDB dataset suggests that our physics-constrained inductive bias facilitates strong generalization across diverse physiological states, ranging from intensive care units to intraoperative anesthesia settings.

A critical factor in realizing this wearable diagnostic potential is the model’s robustness to motion artifacts, while our preprocessing pipeline utilizes standard bandpass filtering (via NeuroKit2) to mitigate basic sensor noise, severe motion artifacts in real-world ambulation often overlap with physiological frequency bands, rendering simple linear filtering insufficient. Fortunately, PhysDiff-LBM provides an inherent, two-fold defense against such disturbances without requiring explicit artifact-simulation training. First, motion artifacts typically present as non-physiological fluctuations that violate cardiovascular fluid dynamics. Because our LBM encoder rigorously enforces mass and momentum conservation, these unphysical noise components are naturally suppressed during the macroscopic moment projection step. Second, the region-disentangled diffusion backbone operates as a strong generative prior. Rather than deterministically mapping noisy inputs to outputs, it guides the reverse diffusion process toward a learned manifold of clean ECG signals. Guided by the topological branch (supervised by the structural ROI masks defined during preprocessing), the model effectively bridges artifact-induced gaps and repairs distortions, preserving both the morphological fidelity and the underlying cardiac rhythm.

Despite these promising advances, several avenues for future research remain. The primary limitation of the current framework lies in the computational cost associated with the iterative sampling process of diffusion models, which poses a challenge for real-time deployment on resource-constrained edge devices. Future work could explore consistency distillation or latent diffusion techniques to accelerate inference speeds without compromising reconstruction quality. Additionally, while this study focuses on single-lead ECG synthesis, extending the LBM-guided framework to reconstruct full 12-lead ECGs from single-point PPG signals remains an open and transformative direction. Such an extension would require incorporating vectorcardiographic constraints to model the spatial propagation of electrical potential, further closing the gap between wearable monitoring and comprehensive clinical cardiology.

## 5. Conclusions

In this paper, we presented PhysDiff-LBM, a novel physics-informed generative framework designed to reconstruct medical-grade ECG signals from ubiquitous PPG recordings. By embedding a differentiable Lattice Boltzmann Method module within a diffusion probabilistic model, we successfully introduced mesoscopic kinetic constraints into the generation process, effectively bridging the domain gap between hemodynamic mechanics and cardiac electrophysiology.

Our extensive experimental evaluation on the MIMIC-III and VitalDB datasets demonstrates that PhysDiff-LBM establishes a new state-of-the-art in reconstruction fidelity, significantly outperforming existing adversarial and regression-based baselines. Crucially, the integration of fluid dynamic principles enables the model to capture the complex non-linear mappings of the cardiovascular system, ensuring precise temporal alignment and morphological consistency even in challenging arrhythmia scenarios. The superior performance observed in downstream clinical tasks, particularly in atrial fibrillation detection, validates the practical utility of our generated signals for automated diagnosis.

Ultimately, this work highlights the potential of synergizing physical laws with generative artificial intelligence. By moving beyond black-box learning to a physics-aware paradigm, PhysDiff-LBM provides a robust and interpretable solution for non-invasive cardiac monitoring, paving the way for accessible, continuous, and reliable cardiovascular healthcare through standard wearable devices.

## Figures and Tables

**Table 1:** Configuration of ablation variants. Checkmarks indicate the presence of each component.

Variant	LBM Encoder	Dilated Streaming	Topology Branch	Cross-Attention
M0: Full Model	✓	✓	✓	✓
M1: w/o LBM Encoder	-	-	✓	✓
M2: w/o Dilated Streaming	✓	-	✓	✓
M3: w/o Topology Branch	✓	✓	-	✓
M4: w/o Cross-Attention	✓	✓	✓	-

**Table 2:** Ablation study results on MIMIC-III and VitalDB datasets. Lower values indicate better performance for all metrics (↓).

Variant	MIMIC-III			VitalDB		
	RMSE ↓	HR-MAE ↓	FD ↓	RMSE ↓	HR-MAE ↓	FD ↓
M0: Full Model	0.22	1.26	6.23	0.25	1.95	4.35
M1: w/o LBM Encoder	0.47	3.85	23.42	0.48	3.92	24.56
M2: w/o Dilated Streaming	0.31	1.82	18.65	0.32	1.88	19.82
M3: w/o Topology Branch	0.26	3.28	10.15	0.27	3.45	11.28
M4: w/o Cross-Attention	0.24	1.75	12.38	0.26	2.12	13.65

**Table 3:** Overview of downstream classification tasks and experimental settings.

Task	Dataset	Network	Classes
Binary Classification for Atrial Fibrillation (AF)	MIMIC PERform AF Dataset	VGG-19	Atrial Fibrillation (AF), Normal Sinus Rhythm (NSR)
Multi-class Arrhythmia Diagnosis	PhysioNet Challenge 2015	VGG-19	Asystole, Bradycardia, Tachycardia, Ventricular Tachycardia (VTA), Ventricular Fibrillation (VFB)

**Table 4:** Downstream clinical classification performance comparison. **Raw PPG** serves as the baseline lower bound. **Bold** text indicates our proposed method (Gen ECG) and the highest performance metrics compared to the baseline. *Italic* text denotes the *Real ECG*, which serves as the ground-truth upper-bound reference (Oracle). Values are reported as Mean ± 95% CI. The improvements of Gen ECG over Raw PPG are statistically significant (p<0.001).

Signal Type	Accuracy	Precision	Recall	F1 Score	AUC
Raw PPG	88.3% ± 1.4%	0.859 ± 0.018	0.949 ± 0.012	0.902 ± 0.016	0.946 ± 0.013
**Gen ECG (Ours)**	**94.7% ± 0.7%**	**0.929 ± 0.009**	**0.981 ± 0.005**	**0.954 ± 0.008**	**0.977 ± 0.006**
*Real ECG (Ref)*	*97.6% ± 0.3%*	*0.971 ± 0.005*	*0.987 ± 0.003*	*0.979 ± 0.004*	*0.998 ± 0.002*

**Table 5:** Multi-class arrhythmia classification performance. **Raw PPG** represents the baseline. **Bold** text indicates our proposed method and the best performance metrics among the generated and baseline signals. *Italic* text denotes the *Real ECG*, which serves as the ground-truth upper-bound reference. 95% Confidence Intervals are provided for the Macro Average results.

Class	Signal	Precision	Recall	F1 Score	AUC
Asystole	Raw PPG	0.700	0.515	0.593	0.795
	**Gen ECG (Ours)**	**0.730**	**0.806**	**0.766**	**0.892**
	*Real ECG (Ref)*	*0.854*	*0.519*	*0.645*	*0.881*
Bradycardia	Raw PPG	0.710	0.832	0.766	0.940
	**Gen ECG (Ours)**	**0.776**	**0.887**	**0.828**	**0.967**
	*Real ECG (Ref)*	*0.752*	*0.903*	*0.821*	*0.959*
Tachycardia	Raw PPG	0.852	0.782	0.816	0.952
	**Gen ECG (Ours)**	**0.869**	**0.925**	**0.896**	**0.976**
	*Real ECG (Ref)*	*0.853*	*0.883*	*0.868*	*0.975*
VF/VFib	Raw PPG	0.703	0.761	0.731	0.915
	Gen ECG (Ours)	0.790	0.671	0.726	0.931
	*Real ECG (Ref)*	*0.775*	*0.900*	*0.833*	*0.948*
VT	Raw PPG	0.684	0.671	0.677	0.824
	Gen ECG (Ours)	0.851	0.657	0.742	0.855
	*Real ECG (Ref)*	*0.854*	*0.650*	*0.738*	*0.892*
**Macro Avg**	Raw PPG	0.730 ± 0.023	0.712 ± 0.027	0.717 ± 0.024	0.885 ± 0.019
	**Gen ECG (Ours)**	**0.803 ± 0.015**	**0.789 ± 0.018**	**0.791 ± 0.016**	**0.924 ± 0.012**
	*Real ECG (Ref)*	*0.818 ± 0.011*	*0.771 ± 0.013*	*0.781 ± 0.010*	*0.931 ± 0.008*

## Data Availability

The datasets used in this article are derived from publicly available datasets. The download links are as follows. MIMIC-III Waveform Database (accessed on 10 January 2026): https://physionet.org/content/mimic3wdb/1.0/; VitalDB (accessed on 10 January 2026): https://vitaldb.net/; MIMIC PERform AF Dataset (accessed on 10 January 2026): https://ppg-beats.readthedocs.io/en/latest/datasets/mimic_perform_af/; PhysioNet Challenge 2015 Dataset (accessed on 10 January 2026): https://archive.physionet.org/physiobank/database/challenge/2015/.

## Abbreviations

The following abbreviations are used in this manuscript:

ECG	Electrocardiogram
PPG	Photoplethysmography
LBM	Lattice Boltzmann Method
N-S	Navier–Stokes
PTT	Pulse Transit Time
ROI	Region of Interest
GAN	Generative Adversarial Network
VAE	Variational Autoencoder
DDPM	Denoising Diffusion Probabilistic Model
PINN	Physics-Informed Neural Network
AF	Atrial Fibrillation
VTA	Ventricular Tachycardia
VFB	Ventricular Fibrillation
RMSE	Root Mean Square Error
FD	Fréchet Distance
